# [^18^F]FET PET Uptake Indicates High Tumor and Low Necrosis Content in Brain Metastasis

**DOI:** 10.3390/cancers13020355

**Published:** 2021-01-19

**Authors:** Hanno S. Meyer, Friederike Liesche-Starnecker, Mona Mustafa, Igor Yakushev, Benedikt Wiestler, Bernhard Meyer, Jens Gempt

**Affiliations:** 1 Department of Neurosurgery, Klinikum rechts der Isar, Technical University of Munich, Ismaninger Str. 22, 81675 Munich, Germany; Bernhard.Meyer@tum.de (B.M.); Jens.Gempt@tum.de (J.G.); 2 Department of Neuropathology, Institute of Pathology, Technical University of Munich, Trogerstr. 18, 81675 Munich, Germany; friederike.liesche@tum.de; 3 Department of Nuclear Medicine, Klinikum rechts der Isar, Technical University of Munich, Ismaninger Str. 22, 81675 Munich, Germany; mona.mustafa@mri.tum.de (M.M.); Igor.Yakushev@mri.tum.de (I.Y.); 4 Department of Neuroradiology, Klinikum rechts der Isar, Technical University of Munich, Ismaninger Str. 22, 81675 Munich, Germany; b.wiestler@tum.de

**Keywords:** brain metastasis, amino acid positron-emission tomography, targeted biopsy, tumor heterogeneity, radiation necrosis, pseudoprogression

## Abstract

**Simple Summary:**

Various types of cancers can lead to brain metastasis. Treatment strategies have improved substantially in the past decade, leading to longer survival in many cases, but also to new diagnostic challenges. Being able to locate those parts of a lesion suspicious for brain metastasis that contain the highest concentrations of viable tumor cells can be crucial, e.g., to obtain a precise diagnosis via targeted biopsies or to differentiate recurring tumor from dead tissue after treatment. Positron emission tomography (PET) imaging has the potential to provide this kind of information. However, studies relating PET findings to actual tissue properties are sparse. The aim of this study was to investigate the association of PET imaging with microscopic tissue properties in samples obtained neurosurgically from brain metastases. Our findings can improve the planning and yield of biopsies from brain metastases, and they may inform future studies aimed at improving the discrimination of recurring from dead tumor in treated brain metastases using PET.

**Abstract:**

Amino acid positron emission tomography (PET) has been employed in the management of brain metastases. Yet, histopathological correlates of PET findings remain poorly understood. We investigated the relationship of O-(2-[^18^F]Fluoroethyl)-L-tyrosine ([^18^F]FET) PET, magnetic resonance imaging (MRI), and histology in brain metastases. Fifteen patients undergoing brain metastasis resection were included prospectively. Using intraoperative navigation, 39 targeted biopsies were obtained from parts of the metastases that were either PET-positive or negative and MRI-positive or negative. Tumor and necrosis content, proliferation index, lymphocyte infiltration, and vascularization were determined histopathologically. [^18^F]FET PET had higher specificity than MRI (66% vs. 56%) and increased sensitivity for tumor from 73% to 93% when combined with MRI. Tumor content per sample increased with PET uptake (r_s_ = 0.3, *p* = 0.045), whereas necrosis content decreased (r_s_ = −0.4, *p* = 0.014). PET-positive samples had more tumor (median: 75%; interquartile range: 10–97%; *p* = 0.016) than PET-negative samples. The other investigated histological properties were not correlated with [^18^F]FET PET intensity. Tumors were heterogeneous at the levels of imaging and histology. [^18^F]FET PET can be a valuable tool in the management of brain metastases. In biopsies, one should aim for PET hotspots to increase the chance for retrieval of samples with high tumor cell concentrations. Multiple biopsies should be performed to account for intra-tumor heterogeneity. PET could be useful for differentiating treatment-related changes (e.g., radiation necrosis) from tumor recurrence.

## 1. Introduction

In advanced neoplastic disease, brain metastases are common, and metastasis is the most frequent type of brain cancer [1]. As diagnostic tools and treatment strategies have improved, the number of cancer patients who are diagnosed with brain metastases has increased. Novel systemic treatment options, such as targeted therapy or immunotherapy, have been able to control intracranial metastases at least in some forms of cancer, and more patients survive longer with brain metastases than before [2]. Long-term follow-up of these patients usually includes MRI with contrast enhancement, which is the cornerstone of diagnostics in brain metastasis. However, it has some limitations, such as low specificity [3,4,5]. This can be problematic in confirming the initial diagnosis as well as during treatment monitoring. Amino acid positron emission tomography (PET), such as O-(2-[^18^F]Fluoroethyl)-L-tyrosine ([^18^F]FET) PET [6], is thought to reveal metastasis-specific uptake of radiolabeled amino acids that is mediated by L-type amino acid transporters [7]. This type of molecular imaging has been well established for primary brain tumors [8], and it has also been proposed to provide useful diagnostic information on brain metastases in addition to MRI imaging [7,9,10]; for a recent review, see [11]. For example, the differentiation of treatment effects (such as post-radiation tissue changes or pseudoprogression following immunotherapy) from tumor recurrence appears to be possible with high specificity and sensitivity, and several studies have found that amino acid PET might even be more useful than MRI for this purpose [12,13,14,15,16]. However, in most of these studies, the histological confirmation of diagnosis and additional imaging were not available for the majority of cases.

Additional clinical applications of amino acid PET are possible. For example, the identification of biopsy targets most likely providing viable tumor tissue yielding a valid diagnosis could be supported by pre-biopsy [^18^F]FET PET imaging, as has been shown for amino acid PET in glioma [17]. This could be helpful not only when histological confirmation is required in suspected newly diagnosed brain metastasis but also when recurrent brain metastasis must be distinguished histologically from post-therapeutic reactive changes, such as radiation necrosis [18]. Thus, even though not recommended by current guidelines, the employment of amino acid PET for biopsy planning might prove useful.

Unlike gliomas, brain metastases are thought to be well-delineated on contrast-enhanced MRI [11]. However, there appears to be a substantial mismatch between tumor volume defined by contrast enhancement and tumor volume defined by [^18^F]FET uptake [19]. It is unclear whether these parts of the metastases differ from those that are congruent in both imaging modalities and whether imaging-based intratumor heterogeneity is also associated with histological intratumor heterogeneity.

To date, little is known about the histopathological changes underlying or accompanying amino acid uptake imaged by PET in brain metastases. In this prospective study, we investigated the relationship between [^18^F]FET uptake (as measured by PET imaging), MRI contrast enhancement, and histological tumor tissue properties using targeted biopsies from brain metastases.

We found that amino acid PET is a helpful diagnostic tool in the management of brain metastases. Amino acid PET in addition to MRI increased sensitivity for tumor in targeted biopsies, and high PET intensity was associated with high tumor and low necrosis content. Consequently, amino acid PET can guide biopsies to parts of the lesion that are more likely to yield high tumor cell concentrations, and it has the potential to differentiate tumor recurrence from treatment-related changes, such as radiation necrosis.

## 2. Results

**Figure 1:**
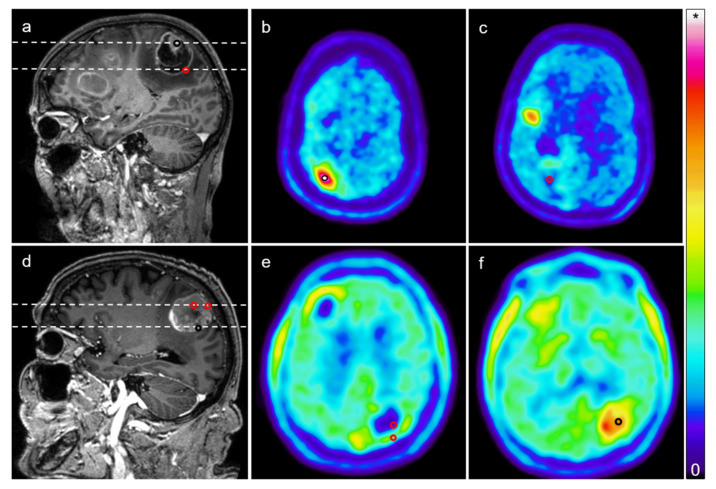
Targeted biopsies from brain metastases. (**a**) Post-gadolinium magnetic resonance imaging (MRI) (T1) showing a contrast-enhancing mass lesion in the right central region of a patient with malignant melanoma; (**b**,**c**) [^18^F]FET positron emission tomography (PET) images corresponding to the imaging planes marked in **a** (upper dashed line: **b**; lower dashed line: **c**) showing elevated tracer uptake in a subvolume of the lesion and low tracer uptake in another part of the lesion. The black circle in a and b indicates the location of a biopsy from a “MRI-positive, PET-positive” part of the lesion that was marked during surgery using intraoperative navigation (mean tumor-to-background ratio (TBR_mean_): 4.27). The red circle in a and c indicates the location of a biopsy corresponding to a “MRI-positive, PET negative” part of the tumor (TBR_mean_: 0.79); (**d**) Another post-gadolinium MRI from a different case (left parietal lesion, adenocarcinoma of the stomach); (**e**,**f**) [^18^F]FET PET images corresponding to imaging planes marked in d (upper dashed line: **e**; lower dashed line: **f**). The black circle indicates the site of a biopsy from a “MRI-positive, PET-positive” part of the lesion, and the red circles indicate “MRI-positive, PET-negative” biopsy sites (TBR_mean_ for anterior red circle: 0.27; posterior red circle: 1.04; black circle: 2.01). Note that MRI contrast enhancement does not necessarily correlate with high tracer uptake, illustrating both PET/MRI mismatch and intratumor heterogeneity. Colorbar illustrates PET standardized uptake values (* maximum in (**b**,**c**): 3.82; (**e**,**f**): 5.0).

All 15 patients (mean age: 59.3 years; range: 45–74 years; eight females, seven males) underwent open resection of one cerebral mass lesion suspicious for metastatic disease. Four of these lesions had been irradiated before. MRI and [^18^F]FET PET imaging were obtained prior to surgery. Virtually all lesions were heterogeneous at the level of imaging, with subvolumes that were PET-positive (i.e., mean tumor-to-background ratio (TBR_mean_) ≥ 1.6 in radiation-naïve lesions and TBR_mean_ ≥ 2.0 in radiated lesions) or negative and MRI-positive (i.e., contrast-enhancing) or negative (Figure 1).

On average, the lesions had a volume of 16.2 ± 11.8 cm^3^ (average ± standard deviation; range: 2.5–41.7 cm^3^). By means of intraoperative navigation, 39 targeted biopsies (two to four per lesion and patient) were obtained from these lesions for the purpose of this study. Two lesions were entirely PET-negative: one was radiation-naïve and had a TBR of up to 1.5; another had been radiated before and had a TBR of up to 1.8. From all other lesions, at least one PET-positive in addition to at least one PET-negative sample was retrieved. MRI-positive samples were obtained from all lesions, and MRI-negative samples were obtained from 11 lesions. Thirteen samples were obtained from the four radiated lesions.

Then, all samples were processed and examined by a neuropathologist. Using tissue from the targeted biopsies and the resection, the diagnosis was confirmed to be metastasized cancer in all cases (origin of neoplasm: four malignant melanomas; four upper/lower gastrointestinal tract carcinomas; four non-small cell lung cancers; one small-cell lung cancer; one breast cancer; one renal cell carcinoma).

**Figure 2:**
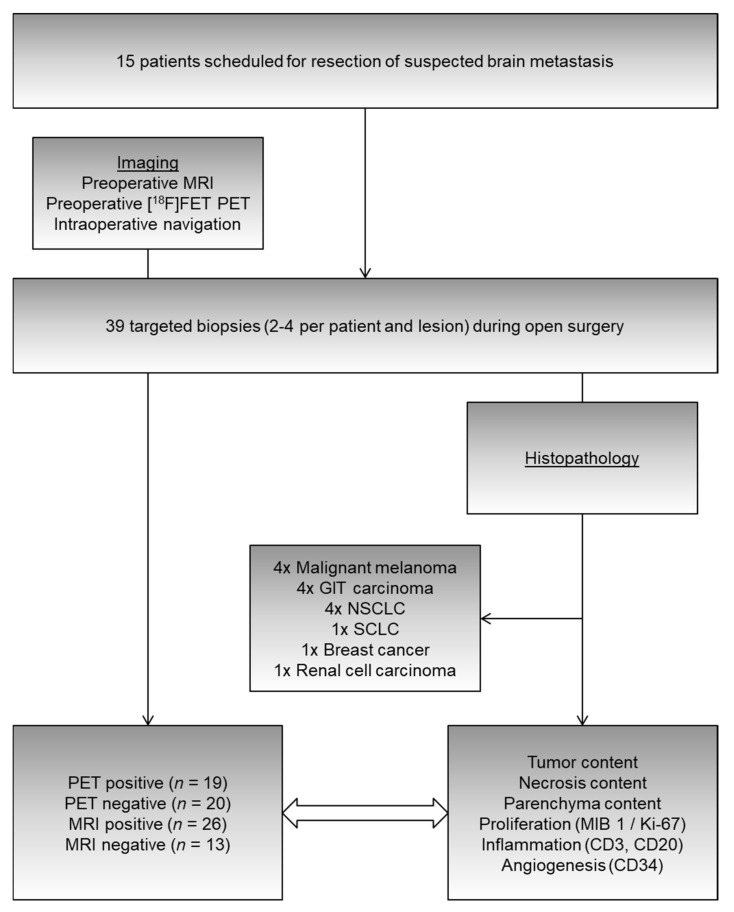
From 15 patients undergoing resection of suspected brain metastases, 39 targeted (i.e., navigated) biopsies were obtained from parts of the tumor that were MRI-positive, MRI-negative, PET-positive, or PET-negative (cf. Figure 1). Histopathological evaluation permitted the investigation of relationships between imaging and tissue properties, such as tumor/necrosis/parenchyma content (percentages), proliferation indices (MIB 1/Ki-67), and markers of inflammation (CD3 = T-lymphocyte infiltration, CD20 = B-lymphocyte infiltration) as well as angiogenesis (CD34). GIT: gastrointestinal tract; NSCLC: non-small cell lung cancer; SCLC: small-cell lung cancer; CD: cluster of differentiation.

To correlate imaging with histological properties, we measured the tumor content, necrosis content, and brain parenchyma content per sample (as percentages) and quantified proliferation as well as the expression of inflammatory and neovascularization markers (Figure 2).

In total, thirty out of the 39 samples contained tumor cells (16 of these were PET-positive; 22 were MRI-positive; 28 were either PET- or MRI-positive). In nine samples, no tumor cells were found. One of these samples contained necrotic material only (it was obtained from a part of the lesion that was both MRI-negative and PET-negative). The remaining eight samples contained parenchyma only. Of these, three were PET-positive, two of which were also MRI-positive. Two of the five PET-negative samples were MRI-positive and three were MRI-negative (MRI: four false positives and five true negatives; PET: three false positives and six true negatives). Consequently, in this setting (i.e., taking samples from substructures of a predefined mass lesion), the specificity for tumor detection was 6/9 = 66% for PET and 5/9 = 56% for MRI (Table 1). Sensitivity was 16/30 = 53% for PET and 22/30 = 73% for MRI. Combining PET and MRI increased the sensitivity to 28/30 = 93%. Ten out of 12 samples from targets that were both PET- and MRI-positive contained a tumor. One out five samples from targets that were both PET- and MRI-negative contained a tumor.

Twenty out of the 26 samples obtained from radiation-naïve lesions contained a tumor. Twelve were PET-positive (60% sensitivity) and 15 were MRI-positive (75% sensitivity). Combined sensitivity was 100%. Both PET and MRI specificity were 3/6 = 50% in these lesions. 

**Figure 3:**
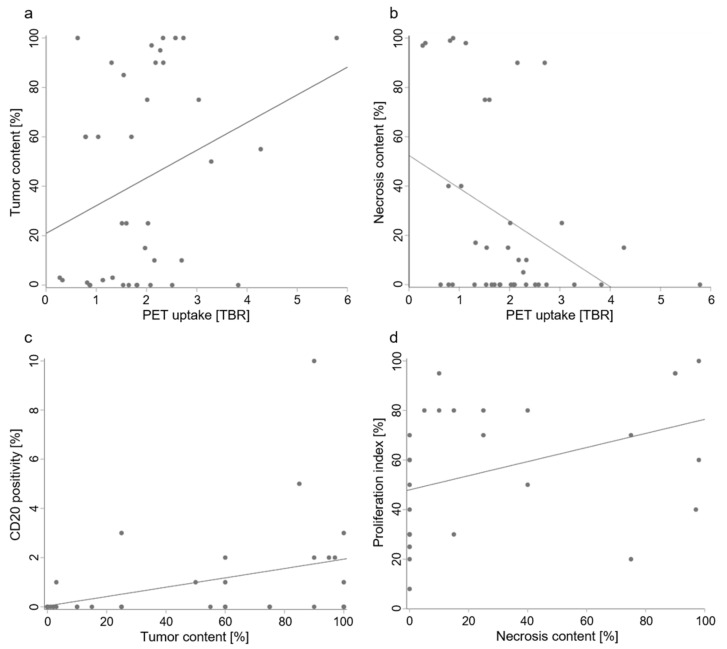
Relationships of PET imaging and histological tumor properties. In brain metastases, ^18^F-FET uptake imaged by PET (measured as mean tumor-to-background ratio (TBR_mean_) of the target volume) increased with tumor content (**a**; r_s_ = 0.3, *p* = 0.045) and decreased with necrosis content (**b**; r_s_ = −0.4, *p* = 0.014). B-lymphocyte infiltration (measured by CD20 positivity) increased with tumor content (**c**; r_s_ = 0.5, *p* = 0.002) and necrosis content increased with proliferation (**d**; r_s_ = 0.5, *p* = 0.002). Note that while scatterplots and linear regression lines are shown to illustrate the data, correlations were analyzed using Spearman’s rank correlation coefficient r_s_, as tumor content, necrosis content, and CD20 positivity were not normally distributed.

There were moderate but statistically significant correlations between PET uptake and tumor content as well as necrosis content per sample (Figure 3): Tumor content increased with PET uptake (r_s_ = 0.3, *p* = 0.045) and necrosis content decreased with PET uptake (r_s_ = −0.4, *p* = 0.014). Moreover, PET-positive samples had higher tumor content than PET-negative samples (*p* = 0.016), with median interquartile range (IQR) tumor percentages of 75 (10 to 97) vs. 3 (0 to 60) %. There was also a trend (*p* = 0.089) toward lower necrosis content with 0 (0–15) % in PET-positive samples vs. 16 (0 to 81) % in PET-negative samples. On the other hand, MRI-positive samples did not differ from MRI-negative samples with regard to tumor or necrosis content (Table 2).

We determined CD3 and CD20 positivity as markers of T-lymphocyte and B-lymphocyte infiltration in samples from brain metastases to investigate potential associations with MRI and PET imaging. Samples from locations with MRI contrast enhancement did not differ significantly from those without contrast enhancement with regard to these markers. There were no significant differences between samples from PET-positive and PET-negative locations either (Table 2), and PET uptake was not significantly correlated with the positivity of these markers. B lymphocyte infiltration increased with tumor content (r_s_ = 0.5, *p* = 0.002; Figure 3).

Ki-67 (MIB 1) positivity in tumor cells as a marker of cell proliferation (proliferation index) did not differ between MRI-positive and MRI-negative samples or between PET-positive and PET-negative samples (Table 3). There was no correlation between proliferation indices and PET uptake. Necrosis increased with proliferation (r_s_ = 0.5, *p* = 0.002; Figure 3).

**Figure 4:**
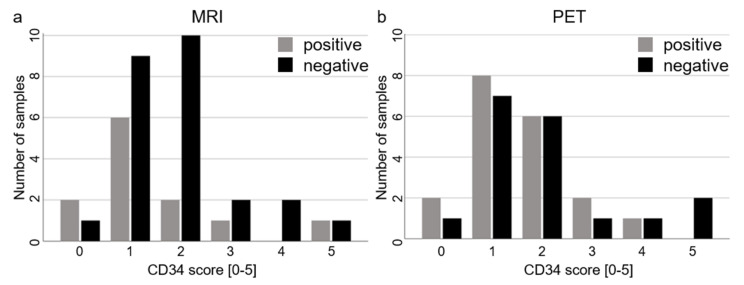
Relationships of PET or MRI imaging and angiogenesis. There were no differences in angiogenesis between MRI-positive and MRI-negative samples (**a**) or between PET-positive and PET-negative samples (**b**), as illustrated by the respective distributions of positive and negative samples sorted by CD34 scores.

Angiogenesis as measured by CD34 expression scores was not significantly correlated with PET uptake, and CD34 scores did not differ significantly between PET-positive and PET-negative or between MRI-positive and MRI-negative samples (Table 2 and Figure 4). Angiogenesis increased with tumor content (r_s_ = 0.4, *p* = 0.009).

Radiation affected cell proliferation. Samples from previously irradiated metastases had significantly lower proliferation indices (39 ± 26 vs. 64 ± 25%, *p* = 0.021). CD3 and CD20 positivity, CD34 scores, and tumor and necrosis contents did not differ.

At the level of histology, tumors were heterogeneous as well. Tumor content per sample, e.g., varied substantially between different specimen from the same lesion (average coefficient of variation (c. v.) per lesion: 60.4%; range of differences between minimum and maximum tumor content per lesion: 0% to 100%). This was also true for the necrosis content (average c. v.: 62.3%; range: 0–100%) and the proliferation index (average c. v.: 21.0%; range: 0–75%).

## 3. Discussion

In the evaluation of brain metastasis, MRI without and with contrast enhancement is the gold standard. Amino acid PET has been proposed to be useful as well, e.g., for the differentiation of radiation-induced changes from tumor progression [11,12,13]. In this study, we investigated histological tumor properties and their relation to [^18^F]FET PET imaging and MRI contrast enhancement in brain metastases.

Our data support that in some clinical situations, additional [^18^F]FET PET imaging can be a helpful tool when treating patients with brain metastases.

Current guidelines do not recommend amino acid PET for biopsy planning, since the size and volume of a metastasis are usually well delineated on contrast-enhanced MRI [11]. We found that [^18^F]FET PET combined with MRI increases sensitivity from 73% to 93% (from 75% to 100% in radiation-naïve lesions) for a tumor that is detected histopathologically, and that tumor content increases with PET uptake. This can be useful when planning targeted biopsies: Upon initial (suspected) diagnosis of possible brain metastases, e.g., primary resection may not be indicated for several possible reasons, such as the location of the lesion, the number of the lesions, the clinical condition of the patient, or the likelihood of differential diagnoses. In these cases, a stereotactic biopsy may be the next diagnostic step. Aiming for a part of the lesion that is not only MRI contrast-enhancing but also has high amino acid PET uptake will increase the chance of obtaining tissue with a high tumor cell density.

Another relevant clinical situation is the suspected recurrence of a previously radiated brain metastasis, where the differential diagnosis of radiation necrosis is a major challenge for treating physicians. It has been suggested that amino acid PET is useful for this purpose, but histological confirmation of the actual diagnosis was sparse in most of these studies [11,12,13,14,15,20,21,22,23,24,25]. For the present study, all samples were evaluated histopathologically, and our findings support the employment of [^18^F]FET PET in addition to contrast-enhanced MRI in these situations. High uptake is in fact correlated with high tumor and low necrosis content, and there is significantly more tumor in [^18^F]FET PET-positive parts of metastases than in [^18^F]FET PET-negative tissue. On the other hand, MRI contrast enhancement was not indicative of high vs. low tumor or necrosis contents. If a biopsy is considered in these patients, it appears to be even more important to aim for targets that are [^18^F]FET PET positive to retrieve samples with high tumor and low necrosis content.

Moreover, based on our findings, amino acid PET-guided biopsies should be considered when molecular testing of tumor tissue is required (or might be required in the future), as the reliability of these tests increases when tumor content is high [26].

It has been shown that brain metastasis-specific uptake of radiolabeled amino acids, such as [^18^F]FET, is mediated by L-type amino acid transporters [27,28] that are commonly overexpressed in brain metastases [7]. We found that both angiogenesis and B-lymphocyte infiltration increased with tumor content, as did [^18^F]FET PET intensity. Necrosis content increased with proliferation and decreased with [^18^F]FET PET intensity. However, neither angiogenesis nor B-lymphocyte infiltration nor proliferation was significantly correlated with [^18^F]FET PET intensity, suggesting that they are not directly linked to the mechanisms underlying [^18^F]FET uptake.

We found that brain metastases are heterogeneous both at the level of imaging and also histologically. Different parts of the same metastasis can be positive and negative for both [^18^F]FET PET and MRI contrast enhancement, and their tumor and necrosis content as well as the proliferation index can also vary substantially. Intratumor heterogeneity can contribute to treatment failure, e.g., due to drug resistance. It has been described at the level of genetic aberrations and gene expression signatures for samples from spatially separated manifestations (primary tumor, distant metastases) of the same neoplasm [29]. Our findings indicate that even within single brain metastases, there may be intratumor heterogeneity. It seems likely that this is not only reflected in imaging and histology variability, but that it is based on variable genetics, protein function, and tumor physiology. This may present a major challenge to personalized medicine and targeted therapies, and any such treatment approach should not rely on single biopsies. Future studies should include genetic and proteomic analyses to further investigate intratumor heterogeneity in brain metastases, and targets of multiple biopsies could be guided by [^18^F]FET PET imaging that might not only be indicative of histological but even molecular intratumor differences.

To minimize bias, we did not exclude small lesions and biopsy targets. It should be noted that this increases a potential targeting error based on the limited imaging resolution as well as the limited precision of the image registration and of the intraoperative navigation. Eight samples turned out to contain parenchyma only. The lesions these samples came from were not significantly smaller than other lesions (13.9 ± 10.5 vs. 17.8 ± 12.9 cm^3^; *p* = 0.55, Student’s *t*-test, unpaired), but in some cases, this may nevertheless be due to targeting errors. This might explain why two samples that were positive for both PET and MRI contained parenchyma only. This would suggest that we underestimated both MRI and PET specificity. However, this could also reflect actual parenchyma contained within the imaging-defined lesions, indicating that brain metastases can grow more infiltrative than often thought [30].

The estimation of tumor cell content as applied in our study (tumor cell percentage) is sensitive to reliability issues [26]. Therefore, for the present study, all samples were evaluated by the same neuropathologist (F.L.S.) to eliminate inter-observer variability.

Another limitation of our study is that although we could not differentiate between different tumor types due to the sample size, not all metastases are the same. It is very likely that some of the associations we found between histology and amino acid PET imaging apply more to some entities than to others. This is, e.g., illustrated by the scatterplots showing tumor and necrosis content vs. PET uptake (Figure 3a,b): even though there is a significant correlation, there appear to be subgroups of samples with virtually no tumor or necrosis that still cover the entire spectrum of PET uptake intensity. The inclusion of metastases that were irradiated before also adds to the heterogeneity of our sample. Future studies need to investigate these issues to even better specify the value of amino acid PET imaging for the management of patients with brain metastases.

## 4. Materials and Methods

This prospective single-center non-interventional study has been approved by the ethics committee of the Klinikum rechts der Isar (project identification code: 160/15). All subjects gave their informed consent for inclusion before they participated in the study. The study was conducted in accordance with the Declaration of Helsinki.

### 4.1. Patients

Patients scheduled for open resection of suspected brain metastases (either solitary metastasis or one out of multiple metastases) based on a recommendation by our multidisciplinary neuro-oncology tumor board were included when they consented to take part in the study.

Exclusion criteria were as follows: contraindication for PET/MRI imaging; contraindication for general anesthesia; indication for emergency surgery prohibiting complete preoperative diagnostic workup; patient declining taking part in the study and/or declining surgery; pregnancy; age below 18 years.

### 4.2. Imaging

All patients underwent [^18^F]FET PET and MRI ahead of surgery. MRI scans were performed on a 3 Tesla (T) MRI scanner, either an Achieva, Ingenia (both by Philips Medical Systems, The Netherlands B.V.) or a Verio (Siemens Healthcare, Erlangen, Germany) device. High-resolution T1-weighted images with and without contrast agent (1mm isotropic) were obtained for all patients. The contrast agent Magnograf^®^ was administered intravenously by a standardized protocol (0.2mL/kg, 0.5–1mL/s) using a MR-compatible contrast medium injection system (Spectris Solaris EP, Siemens Medical, Erlangen, Germany).

[^18^F]FET PET scans were obtained using a Biograph 16 PET/CT (10 patients) or a Biograph mMR PET/MRI (5 patients; both from Siemens Medical Solutions USA, Malvern, PA, USA); note that an MRI was obtained as specified above for all patients independently of PET imaging. Patients were asked to fast for a minimum of 4 h before scanning. A target dose of 185 ± 10% MBq [^18^F]FET was administered intravenously. For PET/CT scans, low-dose CT (24–26 mAS, 120 kV) for attenuation correction was acquired, and 30–40 minutes after injection, PET acquisitions were performed. Static PET data were reconstructed by filtered back-projection using a Hann filter with a cutoff frequency of 0.34 Nyquist into 128 × 128 matrices with a voxel size of 2.1 × 2.1 × 2.4 mm^3^. For PET/MRI scans, static images at 30–40 minutes after injection were reconstructed using 3D OSEM into 192 × 192 matrices with a voxel size of 1.16 mm^3^.

### 4.3. Image Analysis

MRI and PET imaging data were analyzed as reported previously [31]. Briefly, they were imported and fused using the iPlan^®^ cranial surgical planning software (Brainlab, Munich, Germany). [^18^F]FET uptake in the biopsy target volumes was determined by an autocontouring process analogous to a study on [^18^F]FET PET in gliomas [32]. Mean tumor-to-background ratios (TBR_mean_) were calculated by normalizing mean [^18^F]FET uptake in the target volumes to mean uptake in the corresponding unaffected region of the contralateral hemisphere according to previous studies and current guidelines on PET imaging in gliomas [8,32,33]. A TBR_mean_ of 1.6 or higher was defined as PET-positive except for samples from lesions that were irradiated before, where a TBR_mean_ of 2.0 or higher was defined as PET-positive according to previous studies [13,20]. Biopsy sites within lesions suspicious for brain metastasis were defined as MRI-positive when they were contrast-enhancing and otherwise defined as MRI-negative.

### 4.4. Surgery

All patients had surgery for the removal of a cerebral mass lesion that was suspicious for metastasis. Using intraoperative navigation (Curve^®^, Brainlab, Munich, Germany) and the fused imaging data, tissue samples were collected from PET-positive, PET-negative (SUV < 1.6), MRI-positive (i.e., contrast-enhancing), and MRI-negative (i.e., not contrast-enhancing) targets within the lesions. The locations of the samples were marked digitally using intraoperative navigation (Figure 1).

### 4.5. Histopathological Analysis

For histopathological analysis, hematoxylin and eosin (HE) staining was performed to quantify percentages of vital tumor (“tumor content”), necrosis (“necrosis content”), and brain parenchyma (i.e., the ratio of tumor tissue, necrotic tissue, and brain parenchyma to total tissue per sample).

Furthermore, immunohistochemistry was performed. Ki-67 was stained to label proliferating cells. A proliferation index was determined for every biopsy by evaluating the percentage of positive cells compared to all vital tumor cells. Labeling for cluster of differentiation (CD)3 and CD20 was used to mark T lymphocytes and B lymphocytes, respectively. The percentage of positive cells in the area of the whole biopsy tissue was determined. We also labeled for CD34 as a marker for vascularization. A score from 0 to 5 was assigned for every biopsy, with 0 = no vessels, 1 = single vessels, 2 = few vessels, 3 = several vessels, 4 = many vessels, 5 = multitudinous vessels.

For immunohistochemistry, 2 µm thick slides were cut and dried at 76 °C, followed by epitope uncovering in pH 6.0 citrate buffer at 95°C for 30 minutes and H_2_O_2_ incubation. MIB 1/anti-Ki-67 antibody (monoclonal, rabbit, clone: SP6, dilution 1:200; Thermo Fisher Scientific, Waltham, MA, USA), anti-CD3 antibody (monoclonal, rabbit, clone: MRQ-39, dilution 1:500; Cell Marque, Rocklin, CA, USA), anti-CD20 antibody (monoclonal, mouse, clone: L26, dilution 1:500; Dako, Glostrup, Denmark) or anti-CD34 antibody (monoclonal, mouse, clone: QBEnd/10, dilution 1:200; SigmaAldrich, St. Louis, MO, USA) was incubated overnight at 4 °C, followed by incubation in biotinylated secondary anti-rabbit IgG antibody (Vector Laboratories, Burlingame, CA, USA) in a dilution of 1:400. Subsequently, ABC reagent (Vector Laboratories, USA) was incubated for 30 min, followed by diaminobenzidine reagent (Dako, Glostrup, Denmark). For all immunostainings, counterstaining with hematoxylin was conducted. Positive controls served as quality assurance.

### 4.6. Statistics

Statistical analysis was performed using IBM SPSS Statistics version 25.0 (SPSS Inc., IBM Corp., Armonk, NY, USA). To compare PET-positive vs. negative and MRI-positive vs. negative as well as irradiated vs. radiation-naïve groups of biopsy samples, we tested the dependent variables (proliferation index, CD3 expression, CD20 expression, CD34 score, necrosis content, tumor content) for normality (Shapiro–Wilk test). All except for the proliferation index were not normally distributed, neither when regarding the entire sample nor when sorted by PET- or MRI-positive vs. negative or by irradiated vs. radiation-naïve. Thus, a Mann–Whitney U test was applied when groups were compared (except for comparing proliferation indices, where unpaired *t*-tests were applied), and correlations were analyzed using Spearman’s rank order correlation. Accordingly, we refer to the median and interquartile range (IQR) when comparing groups with regard to non-normally distributed variables (Table 2). Means and standard deviations (SD) are reported as well but should be interpreted with caution for all variables except for the proliferation index (Table 3). An error probability of less than 0.05 was considered statistically significant.

## 5. Conclusions

In this study, [^18^F]FET PET provided clinically valuable information on brain metastases. PET increased sensitivity for histopathologically detectable tumor from 73% (MRI contrast enhancement alone) to 93% (MRI and PET combined). Higher uptake indicated higher tumor and lower necrosis content. This confirms that in brain metastasis treatment, amino acid PET can be helpful for the differentiation of actual tumor progression from treatment-associated (necrotic) tissue changes, i.e., pseudoprogression. Moreover, this argues for the application of amino acid PET-guided selection of biopsy targets.

[^18^F]FET PET and targeted biopsies revealed intratumor heterogeneity in brain metastases. This should be investigated at the molecular level in future studies and suggests that if possible, multiple biopsies should be acquired from single metastases.

## Figures and Tables

**Table 1:** Error matrix for PET, MRI, and combined PET/MRI imaging.

Imaging Property	Tumor in Specimen (*n* = 30)	No tumor in Specimen (*n* = 9)
PET//MRI Positive	16//22 (combined: 28)	3//4 (combined: 5)
PET//MRI Negative	14//8 (combined: 2)	6//5 (combined: 4)

**Table 2:** PET- or MRI-positive vs. negative samples (median, interquartile range).

Histological Property	PET-Positive	PET-Negative	MRI-Positive	MRI-Negative
Tumor content (%)	75 (10–97)	3 (0–60)	60 (3–90)	10 (0–50)
Necrosis content (%)	0 (0–15)	16 (0–81)	13 (0–36)	0 (0–75)
Proliferation index (%)	60 (30–80)	60 (30–70)	65 (30–80)	50 (25–65)
CD20 expression (%)	0 (0–2)	0 (0–0.5)	0 (0–1)	0 (0–2)
CD3 expression (%)	1 (1–4)	1 (0–3)	1 (0–2)	3 (1–5)
CD34 score	2 (1–2)	1 (1–2)	2 (1–2)	1 (1–2)

**Table 3:** PET- or MRI-positive vs. negative samples (mean, standard deviation).

Histological Property	PET-Positive	PET-Negative	MRI-Positive	MRI-Negative
Tumor content (%)	60 ± 40	27 ± 35	50 ± 41	28 ± 36
Necrosis content (%)	14 ± 28	39 ± 42	26 ± 35	28 ± 44
Proliferation index (%)	58 ± 28	54 ± 27	58 ± 28	51 ± 28
CD20 expression (%)	1 ± 2	1 ± 1	1 ± 2	1 ± 1
CD3 expression (%)	3 ± 5	3 ± 4	3 ± 5	3 ± 3
Tumor content (%)	60 ± 40	27 ± 35	50 ± 41	28 ± 36

## Data Availability

Most of the data presented in this study are available in the article. Some data, such as complete MRI imaging sets, are not publicly available due to data protection regulations.
